# The sodium-glucose co-transporter 2 inhibitor velagliflozin reduces hyperinsulinemia and prevents laminitis in insulin-dysregulated ponies

**DOI:** 10.1371/journal.pone.0203655

**Published:** 2018-09-13

**Authors:** Alexandra Meier, Dania Reiche, Melody de Laat, Christopher Pollitt, Donald Walsh, Martin Sillence

**Affiliations:** 1 Earth, Environmental and Biological Sciences School, Queensland University of Technology, Brisbane, Queensland, Australia; 2 Boehringer Ingelheim Vetmedica, Ingelheim am Rhein, Germany; 3 Australian Equine Laminitis Research Unit, School of Veterinary Science, University of Queensland, St Lucia, Queensland, Australia; 4 Animal Health Foundation, Pacific, Missouri, United States of America; Massey University, NEW ZEALAND

## Abstract

There are no registered veterinary drugs for treating insulin dysregulation and preventing insulin-associated laminitis in horses. Velagliflozin is a sodium-glucose co-transport 2 inhibitor that reduces renal glucose reabsorption, promotes glucosuria, and consequently, decreases blood glucose and insulin concentrations. This study aimed to determine if velagliflozin reduced hyperinsulinemia and prevented laminitis in insulin-dysregulated ponies fed a challenge diet high in non-structural carbohydrates (NSC). An oral glucose test (1 g dextrose/kg BW) was used to screen 75 ponies for insulin dysregulation, of which 49 ponies with the highest insulin concentrations were selected. These animals were assigned randomly to either a treated group (n = 12) that received velagliflozin (0.3 mg/kg BW, *p*.*o*., *s*.*i*.*d*.) throughout the study, or a control group (n = 37). All ponies were fed a maintenance diet of alfalfa hay for 3 weeks, before transferring to a challenge diet (12 g NSC/kg BW/d) for up to 18 d. Blood glucose and serum insulin concentrations were measured over 4 h after feeding, on d 2 of the diet. The maximum glucose concentration was 22% lower (P = 0.014) in treated animals, with a geometric mean (95% CI) of 9.4 (8.0–11.0) mM, versus 12.1 (10.7–13.7) mM in the controls. This was reflected by lower (45%) maximum insulin concentrations in the treated group (P = 0.017), of 149 (97–228) μIU/mL, versus 272 (207–356) μIU/mL for controls. The diet induced Obel grade 1 or 2 laminitis in 14 of the 37 controls (38%), whereas no velagliflozin-treated pony developed laminitis (P = 0.011). Velagliflozin was well-tolerated, with no hypoglycemia or any clinical signs of adverse effects. The main limitation of this study was the sample size. Velagliflozin shows promise as a safe and effective compound for treating insulin dysregulation and preventing laminitis by reducing the hyperinsulinemic response to dietary NSC.

## Introduction

Laminitis is a common and painful condition of the horse’s foot, causing lameness, and in severe cases, death from euthanasia. Endocrinopathic laminitis, associated with insulin dysregulation and hyperinsulinemia, or pituitary *pars intermedia* dysfunction (PPID), is thought to be the most prevalent form of the disease [1].

The cause of hyperinsulinemia is multi-factorial, but the ingestion of a diet that is high in non-structural carbohydrates (NSC) by animals that have tendency for enhanced glucose absorption has been shown to be a likely factor [2]. In contrast to other species, horses rarely show marked hyperglycemia or suffer glucose toxicity, as the equine pancreas can produce large amounts of insulin for prolonged periods, without pancreatic failure [3]. This can result instead in insulin toxicity, which is manifest by laminitis—a failure of the suspensory apparatus or ‘lamellae’ connecting the distal phalanx to the hoof wall [4].

Therapeutic options for treating hyperinsulinemia and preventing laminitis are limited, with no veterinary pharmaceutical treatments currently registered. In cases of hyperinsulinemia with concurrent PPID, pergolide mesylate reduces hyperinsulinemia in some animals, but does not cause this effect consistently [5]. Husbandry strategies can reduce laminitis risk, including restricting access to pasture and a low NSC diet [6]. Exercise can also improve insulin sensitivity in obese equids [7,8] and light regular exercise is sufficient to maintain this improvement [9]. However, there are circumstances where pharmacological intervention would be helpful.

Sodium-glucose cotransport 2 (SGLT-2) inhibitors are a class of human anti-diabetic drugs that offer a novel therapeutic option for treating insulin dysregulation and hyperinsulinemia in horses through targeting renal glucose reabsorption. The kidney has a key role in regulating blood glucose concentrations by mediating the reabsorption of glucose back into plasma following filtration of the blood. The SGLT proteins are responsible for glucose reabsorption, with SGLT-2 performing approximately 90% of reabsorption, while SGLT-1 facilitates the remainder [10,11]. SGLT-2 inhibitors block this renal transporter, promoting glucosuria and reducing blood glucose concentrations, which consequently reduces insulin concentrations as a compensatory physiological response.

This study aimed to test the hypothesis that the SGLT-2 inhibitor velagliflozin attenuates the hyperinsulinemic response in insulin-dysregulated ponies fed a diet high in NSC, and in doing so, decreases the incidence of laminitis in these susceptible animals.

## Materials and methods

### Animal selection, characteristics and management

This study was approved by the Animal Care and Ethics Committees of the University of Queensland (Approval # QUT/SVS/114/14) and Queensland University of Technology (Approval # 1400000575) and was carried out strictly in accordance with the approval. The study design was a randomized controlled trial and the full details of animal selection, management and experimental procedures described here have been reported previously [12]. Briefly, 75 ponies were purchased from local owners and dealers and screened with a standardized Oral Glucose Test (OGT) to assess insulin dysregulation by feeding 1 g dextrose/kg BW (Body weight; Sigma-Aldrich, Castle Hill, Australia) mixed with 200 mL tap water, 0.15% BW lucerne (alfalfa) chaff and 200 g wheat bran [13]. Blood samples were collected by jugular venipuncture at 0800 h before the meal (fasting) and 2 h later, for the analysis of blood glucose and serum insulin concentrations. Forty-nine ponies with the highest 2 h insulin concentration were enrolled, and allocated at random to either a control (n = 37) or treated group (n = 12).

Due to the limited number of available stables, ponies entered the study over 12 months in eight individual groups ranging in size from four to nine ponies per group. Each group received identical treatment throughout the study including pre-study health-management procedures, housing, diet and handling, as described previously in detail, and in brief here [12]. Randomization within these groups was achieved by pre-determining the order of treatment allocation, then assigning each pony to the treated or control group according to this list and their order of presentation (ID number). During the selection phase, the ponies were housed in small groups in yards and fed lucerne hay (1.8% BW) divided into two daily meals, plus a vitamin/mineral supplement (Equilibrium mineral mix, Loganholme, Australia) mixed with 200 g lucerne chaff. All ponies were subjected to standard health-management procedures, including Hendra virus vaccination, dental and anthelmintic treatment and hoof-trimming. Mares were pregnancy tested by ultra-sound and only enrolled if non-pregnant. Basic training and handling familiarised ponies with the experimental procedures while they adjusted to their environment. A thorough veterinary health assessment was performed at the start and the end of the experimental period, including the measurement of blood biochemistry, hematology and basal adrenocorticotrophic hormone (ACTH) concentrations. The clinical examination included: BW, body condition score (BCS), cresty neck score (CNS), demeanor, thoracic and abdominal auscultation, heart rate, respiratory rate, temperature, forelimb digital pulse palpation, capillary refill time, mucous membrane colour, skin turgor, lymph node palpation, lameness examination, and visual inspection for any abnormalities.

The ponies were a mix of eight breeds and derivatives, dominated by Shetland Pony, Miniature Horse, Welsh Mountain Pony, Connemara, Arabian and Australian Pony breeds. Body condition score was allocated on a scale of 1 to 9 [14] and CNS from 1 to 5 [15]. The two groups had similar morphometric characteristics, although the treated group was younger in age (Table 1).

**Table 1:** Age and morphometric measures (median, IQR or mean ± SE) in two randomly allocated groups of ponies.

Measure	Control (n = 37)	Treated (n = 12)	P value^a^
Age (yr)	15 (10.5–18.5)	10.5 (8.3–13)	0.022
Wither height (in)	40 (33.5–49)	40.5 (34.5–50.5)	0.904
Weight (kg)	210 ± 13	219 ± 27	0.820
Body condition score	7.1 ± 0.2	7.5 ± 0.3	0.358
Cresty neck score	3.8 ± 0.1	4.0 ± 0.3	0.474
Sex	16 M, 21 F	7 M, 5 F	0.508

^*a*^ Following a D’Agostino and Pearson test for normality, *p* values were calculated using an un-paired Student’s t-test or a Mann Whitney U-Test, as appropriate, except for gender composition, which was compared using Fisher’s exact test.

### Experimental procedures

During the experimental phase, the ponies were housed in individual stables on sawdust substrate, and provided with 2 h daily exercise outside in pairs. Treated ponies were dosed with velagliflozin (0.3 mg/kg BW *p*.*o*.) for 21 d prior to, and for the 18-d of the diet challenge period (DCP). The velagliflozin powder (Boehringer Ingelheim Vetmedica GmbH, Ingelheim, Germany) was dissolved and diluted in an aqueous carrier such that each pony received 0.06 mL of the solution per kg BW, delivered by oral syringe once daily at 0800 h. The dose rate of velagliflozin was determined by Boehringer Ingelheim Vetmedica GmbH after pharmacokinetic and pharmacodynamic studies in horses and other species (unpublished data).

An OGT was performed to assess insulin dysregulation status in ponies allocated to the treatment group within 5 d before starting velagliflozin and in control ponies within 5 d before starting the diet challenge period (DCP). In addition, all ponies were examined to confirm their soundness, including a clinical laminitis examination, hoof radiographs with measurement of the angle of rotation from distal phalanx parietal surface to the dorsal hoof wall, and digital pulse palpation; full details are described elsewhere [12].

During the DCP, the ponies were fed three daily meals at 0800 h, 1200 h and 1600 h of a mixed-feed each containing 2.7 g/kg BW roasted, micronized oat flakes (Riverina Stock Feeds, Milton, Australia), 2.7 g/kg BW molasses, 1.7 g/kg BW lucerne chaff, and 0.3 g/kg BW dextrose. A vitamin/mineral supplement (Equilibrium mineral mix, Loganholme, Australia) was added to the 1600 h meal (dosed by BW according to manufacturer recommendations). Lucerne hay (5 g/kg BW) was fed at 1630 h to provide extra roughage for intestinal health. The diet contained 36.6% roughage overall, and provided ~ 12 g NSC/kg BW daily. To allow for gastrointestinal adjustment, one meal of the mixed-feed was given at 0800 h daily for 3 d before the DCP, plus 1.8% BW lucerne hay. The DCP continued for up to 18 d, or less if a pony developed laminitis, in which case the diet was stopped immediately and replaced by grass hay. Feed refusals were weighed at 0800 h daily, faecal consistency was monitored twice daily, and faecal pH was measured as described [12]. Laminitis was diagnosed from video analysis by two blinded experts, and this method has also been described previously [12].

On the evening of the first day of the DCP, jugular catheters were aseptically fitted. On d 2, a blood sample was collected at 0800 h (0 min), the drug or placebo was administered and the ponies fed their first meal of the day. More blood samples were collected after 60, 90, 120 and 240 min to determine the insulin and glucose response to the challenge diet. Feed refusals were weighed at the 240 min sample point to measure diet consumption over the blood sampling period.

Blood sampling and assay procedures for the measurement of insulin, glucose and ACTH concentrations, including validation data for these assays, have been described previously [12].

### Data analysis

The data were subjected to the D’Agostino and Pearson or a Shapiro-Wilk test for normality. Data that did not conform to a normal distribution were log transformed and re-tested. If a normal distribution was still not achieved, then the data were analysed using the non-parametric Wilcoxon, Mann-Whitney U and Kruskal-Wallis (with post-hoc Dunn’s test) tests, and are presented as medians (inter-quartile range, or total range).

Parametric data were analysed using a paired or unpaired Student’s t-test for 2-way comparisons. Multiple groups were compared using Analysis of Variance (ANOVA), with or without repeated measures. Means were separated using Tukey’s post hoc test. The results are reported as mean ± SE for normal data, or geometric mean (95% confidence interval) for data that were normalized after transformation. Fisher’s exact test was used to determine differences between control and treated groups when assessing the binary (yes/no) outcome of gender; elevated ACTH concentrations; PPID diagnosis; and frequency of laminitis according to PPID diagnosis. These analyses were made using Prism 7 software (GraphPad Software, La Jolla, CA, USA). Significance was set at P < 0.05.

## Results

### Oral glucose test

The pre-DCP data show a relatively uniform distribution of insulin and glucose responses to the OGT across the ponies allocated to the control and treatment groups, with no significant difference between the groups for any variable measured (Table 2).

**Table 2:** Results from an oral glucose test performed prior to feeding a diet high in non-structural carbohydrates in a laminitis prevention study^a^^,^^b^.

	Control (n = 37)	Treated (n = 12)^c^	P-value
0 h Glucose, mM	4.8 (4.5–5.3)	4.8 (4.1–5.3)	0.593
2 h Glucose, mM	9.6 (7.8–11.5)	10.8 (8.3–14.2)	0.271
0 h Insulin, μIU/mL	6 (4–10)	6 (2–18)	0.886
2 h Insulin, μIU/mL	80 (54–170)	128 (56–187)	0.487

^a^Data are expressed as median (IQR).

^b^Values were compared using a Mann Whitney U-Test.

^c^Testing was performed with 5 d prior to velagliflozin commencing

### Laminitis

**Fig 1:**
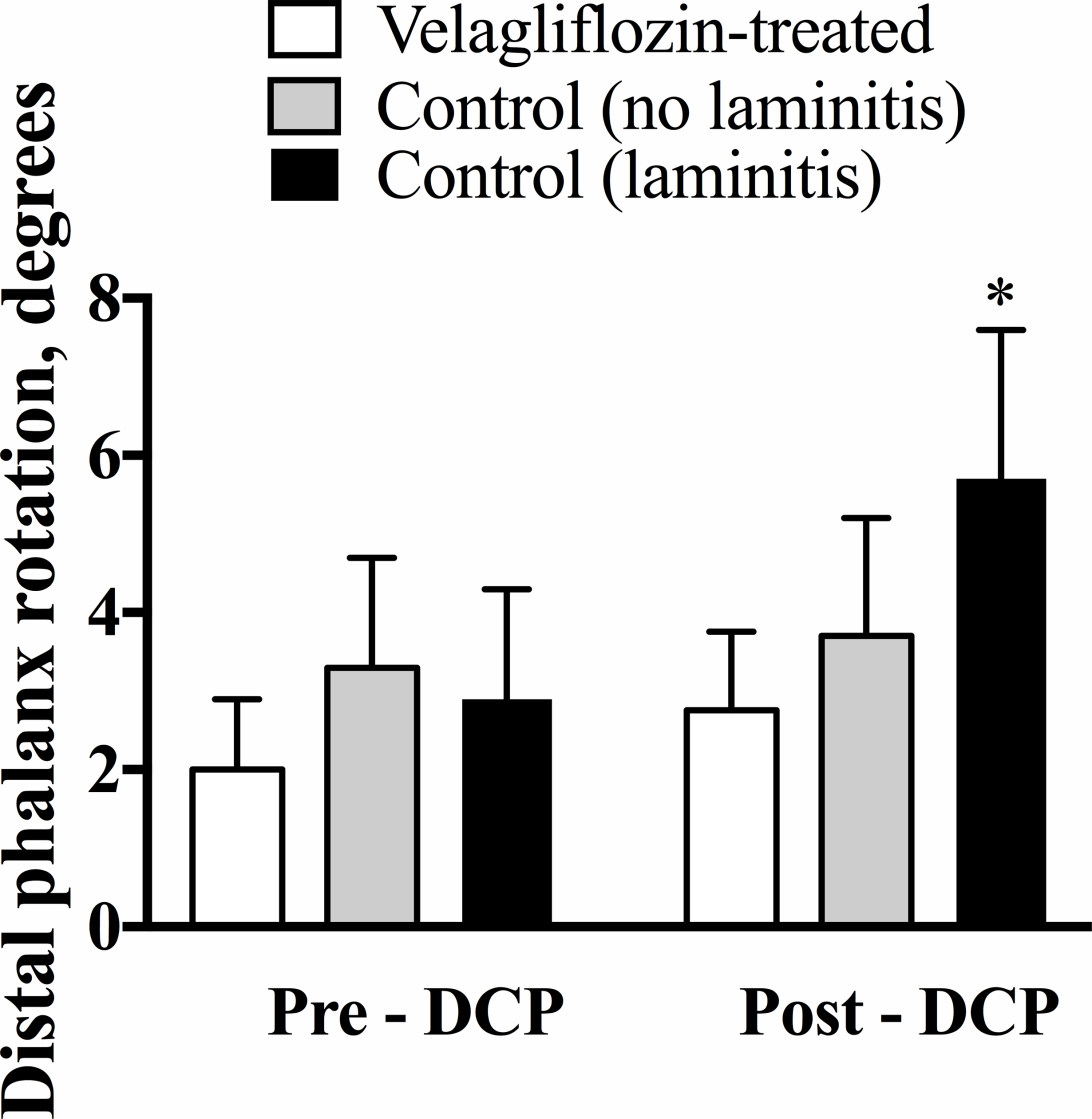
Average rotation of the distal phalanx (degrees) taken from both front feet in ponies before and after a diet challenge period (DCP). *P = 0.01 for comparison between pre- and post-diet challenge values using a paired Student’s t-test.

None of the 12 velagliflozin-treated ponies developed clinical laminitis, in contrast to 14 of the 37 control ponies (38.5%) that developed clinical laminitis as diagnosed by two experts blinded to each other’s diagnosis [12]. Thus, the frequency of laminitis observed between the two groups was significantly different (P = 0.011). The data for laminitis examinations are shown in S1 Table. In addition, pre-DCP radiographs were similar between all groups of animals (P = 0.734), whereas the ponies that developed laminitis showed increased rotation of the distal phalanx (P = 0.01), as measured on the day of laminitis diagnosis (Fig 1).

### Dietary glucose and insulin response

Glucose and insulin responses were measured on d 2 of the DCP in all animals, but the data for eight control ponies have been excluded from the analysis due to their failure to consume more than 50% of their morning ration during the 4-h measurement period. Ponies included in the data analysis consumed on average 93% of the meal.

Velagliflozin reduced both the insulinemic and glycemic response to the challenge diet. The maximum insulin concentration (C_max_) over the 4 h of testing was 45% lower for the velagliflozin-treated group compared to all controls (P = 0.017), with a geometric mean (95% CI) of 149 μIU/mL (97–228), in comparison to 272 μIU/mL (207–356). The C_max_ for glucose followed the same pattern, and was 22% lower (P = 0.014) in treated animals (9.4 mM, 8.0–11.0), compared to all controls (12.1 mM, 10.7–13.7). Baseline 0800 h insulin concentrations (0 min DCP sample) also showed a trend towards being lower in treated ponies (P = 0.063), with a median (IQR) value of 8 μIU/mL (4–95) compared to 52 μIU/mL (10–189) for controls. Importantly, baseline glucose concentrations were not different between groups (P = 0.697) with a median (IQR) of 4.9 mM (4.4–6.1) in the controls and 5.0 mM (4.7–6.2) in treated ponies. Additionally, the lowest glucose concentration recorded for any treated pony was 4.1 mM, and no hypoglycemia occurred.

Separating the controls into two groups (laminitis or no laminitis) and comparing these to the velagliflozin-treated group revealed a pronounced difference between the three groups in terms of their glucose and insulin responses. As shown in Table 3, the glucose C_max_ in response to the dietary challenge was 37% lower in the velagliflozin-treated group than in laminitis-positive controls (P < 0.001), but was not different to the C_max_ in controls that didn’t develop laminitis (P = 0.380). The insulin C_max_ followed the same pattern, and was 62% lower in the velagliflozin-treated group than in laminitis controls (P = 0.003), but was not different in comparison to the non-laminitic controls (P = 0.296).

**Table 3:** Maximum blood glucose and serum insulin concentrations (geometric mean and 95% CI) measured over 4 h after feeding a meal high in non-structural carbohydrates comparing control ponies that either did or did not develop laminitis, to velagliflozin-treated ponies, none of which developed laminitis.

	Control—laminitis positive (n = 11)^a^	Control—no laminitis (n = 18)^a^	Velagliflozin-treated (n = 12)
Glucose, mM	14.9 (12.9–17.2)^b^	10.7 (9.2–12.5)^c^	9.4 (8.0–11.0)^c^
Insulin, μIU/mL	396 (301–520)^b^	216 (148–316)^c^	149 (97–228)^c^

^a^Eight control animals were excluded from the analysis due to their failure to consume more than 50% of the meal within 4 h of feeding.

^b,c^Means in a row with different superscripts, P < 0.05, Kruskal-Wallis test with post-hoc Dunn’s test.

**Fig 2:**
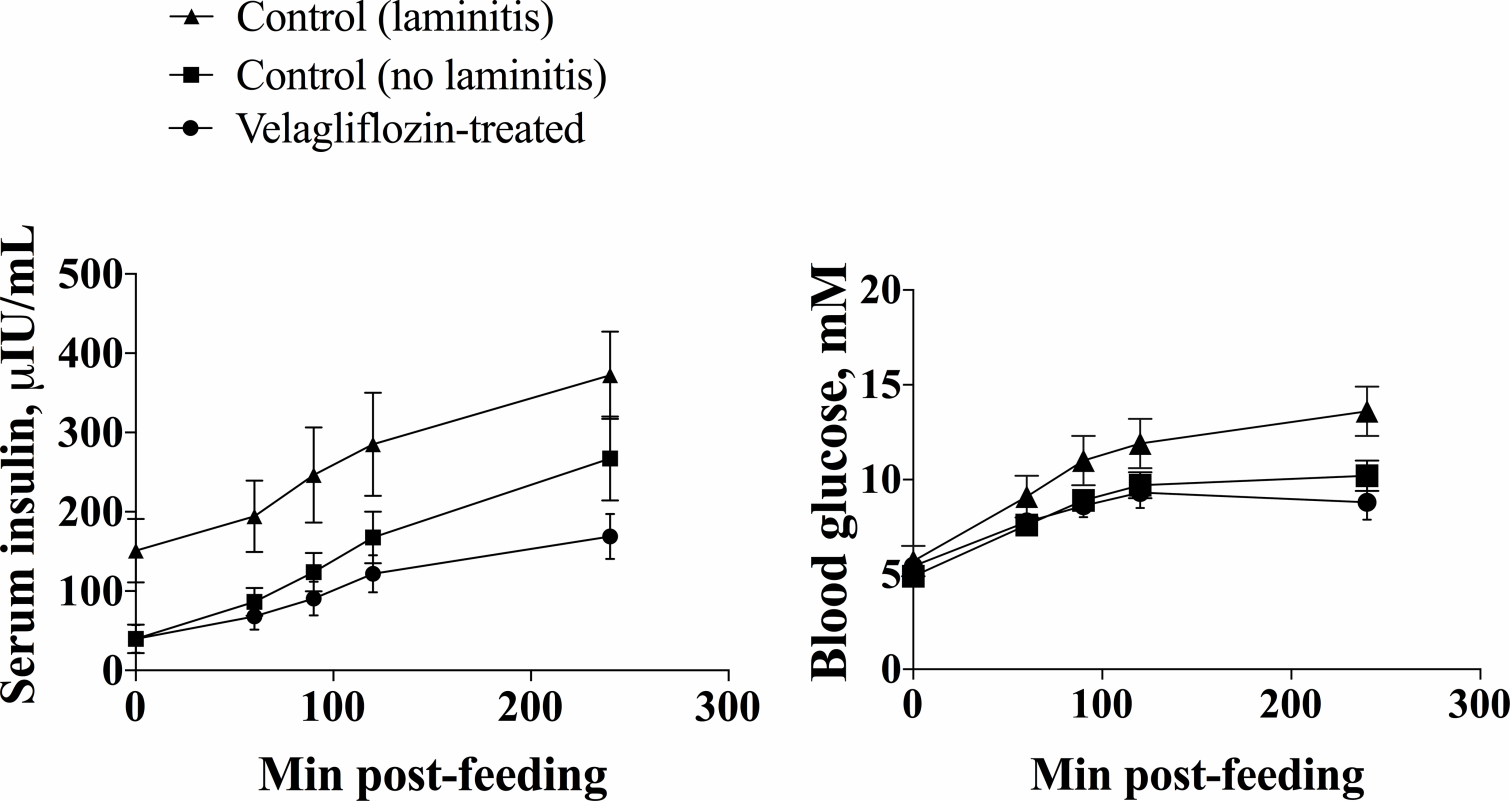
Geometric mean (95% CI) concentrations of insulin (A) and glucose (B) measured at 0, 60, 90, 120 and 240 min post-feeding on the second day of a diet challenge period in control ponies that developed laminitis (n = 11), control ponies that did not develop laminitis (n = 18) and velagliflozin-treated ponies (n = 12).

In these same three groups (control-laminitis positive, control-no laminitis and velagliflozin-treated) the insulin and glucose log transformed data collected over the 4 h challenge test was subjected to two-way repeated measures ANOVA, confirming an effect of both time (P < 0.0001) and treatment (P = 0.002) for glucose; and time (P < 0.0001) and treatment (P < 0.0001) for insulin; these data are shown in S2 Table. Fig 2 demonstrates that the glucose and insulin response was highest in the control-laminitis group, and was similar between the control-no laminitis and velagliflozin-treated groups.

### Feed intake

Feed refusals were not significantly different between the three groups over the entire 18 d DCP (P = 0.320), with mean ± SE daily feed refusal of 5.2 ± 1.5%, 3.2 ± 1.3% and 0.96 ± 0.5% for controls that did not develop laminitis, controls that did develop laminitis, and velagliflozin-treated ponies.

### Pituitary *pars intermedia* dysfunction

Pre-experimental basal ACTH concentrations were above the seasonally-adjusted normal range for the Southern hemisphere [16] in 16/37 control ponies (43%), and in 7/12 ponies in the treated group (58%), with this frequency not significantly different between groups (P = 0.508). Of the controls, six had elevated ACTH in non-autumn months, with a median (range) concentration of 54 (30–96) pg/mL, and ten were elevated in Autumn, with a concentration of 175 (103–1250) pg/mL. Of the animals allocated to the treatment group, one was elevated in summer with a concentration of 364 pg/mL, and 6 were elevated in autumn, with a median (range) concentration of 319 (78–1073) pg/mL.

However, the actual diagnosis of PPID was based on a combination of elevated ACTH concentrations above the reference ranges, plus clinical signs of PPID including hirsutism and polydipsia/polyuria. Using these criteria, five controls and four treated ponies were diagnosed with PPID, giving a frequency that was also not significantly different between groups (P = 0.195). Importantly though, the frequency of laminitis with concurrent PPID was different between the control and treated groups (P = 0.008), as all five controls with concurrent PPID developed laminitis, whereas none of the four velagliflozin-treated ponies with PPID developed laminitis. Neither group was treated with pergolide mesylate either before or during the study.

### Acceptance and tolerability of velagliflozin

Velagliflozin was easy to administer and was well accepted orally. Of the 12 treated ponies, no adverse reaction was observed in any subject and no ponies were withdrawn from the medication during the 39 d treatment period. No abnormalities were detected in routine biochemistry and hematology measurements performed both before and after treatment, (data are presented in S3 Table.

### Body weight and condition

Over the 39 d experimental period, from the start of drug treatment to the end of the DCP, the 23 control ponies that did not develop laminitis showed a 3% increase (P < 0.0001) in total BW (mean ± SE) from 215 ± 19 kg to 221 ± 20 kg. Whereas the 12 velagliflozin-treated ponies had no significant change from 219 ± 27 kg to 215 ± 25 kg (P = 0.216). The BCS and CNS values did not change significantly within or between these two groups either, except for one instance where an increase in CNS (geometric mean, 95% CI) was seen in the 23 non-laminitic controls from 3.7 (3.3–4) at the study beginning to 4.1 (3.8–4.5) at the finish (P = 0.002). Bodyweight and condition data are shown in S4 Table. Laminitis cases were excluded from BW and body condition analysis due to finishing the DCP before the other horses upon the development of laminitis.

## Discussion

This study has demonstrated that velagliflozin can reduce the glucose and insulin response of ponies fed a high NSC diet, and that it decreases the frequency of laminitis when given to insulin-dysregulated ponies daily for 21 d before, and for 18 d during, a high NSC diet challenge. The study is the first of its kind in demonstrating the prevention of insulin-associated laminitis using a pharmacological agent, and supports further clinical testing of this compound. It is especially encouraging that velagliflozin was readily accepted and well tolerated by all 12 ponies over the 39 d treatment period.

With no adequate treatments for insulin-associated laminitis in horses, the focus must be on both the early identification of at-risk individuals, and the prevention of laminitis [17]. SGLT-2 inhibitors could address this issue in terms of laminitis prevention, and provide a novel treatment option for improving insulin dysregulation. In humans, SGLT-2 inhibitors are used in the treatment of type 2 diabetes, and have been shown consistently to reduce blood glucose concentrations and improve insulin sensitivity without causing hypoglycemia [18,19]. The current study mirrors results from the human field, and provides evidence to support the use of velagliflozin as a long-term laminitis preventive agent in insulin-dysregulated equids.

Interestingly, laminitis was not only prevented in insulin-dysregulated ponies, but also in ponies diagnosed with clinical PPID, four of which were included in the treatment group. In comparison, the five ponies in the control group with clinical PPID all developed laminitis (P = 0.008). No pony in either group was treated with pergolide mesylate, and to our knowledge, none had ever received treatment for PPID. The fact that no velagliflozin-treated pony developed laminitis despite concurrent clinical PPID is very encouraging for the compound, but also supports prior studies showing that the likelihood of laminitis occurring in PPID animals can be directly related to the degree of insulin dysregulation [20–22]. These results support the possibility of a relationship, previously described as an ‘endocrinopathic convergence’, between insulin dysregulation and the dopaminergic neurodegeneration resulting in PPID, although this relationship is complicated by the fact that not all horses with PPID have insulin dysregulation [23].

As well as improving insulin and glucose dynamics, other potential benefits of velagliflozin beyond those considered in this study could include improvements in lipid profiles or BW, as triglyceride concentrations have been shown to decrease with long-term treatment [24]. Elevated triglyceride concentrations occur in previously laminitic, obese and hyperinsulinemic [25–27] horses, and this aspect deserves further investigation in horses. In addition, the effect of velagliflozin on BW should be investigated further as the current study was too short to accurately determine the effects on BW and body condition. The role of obesity in laminitis is unclear, but as insulin concentrations correlate positively with obesity [28–30], weight loss as a secondary benefit of SGLT-2 inhibition could be desirable in certain cases.

Alongside the potential benefits of velagliflozin treatment, the potential risks should be considered. An obvious concern about glucose-lowering treatments is the risk of hypoglycemia. In the present study, the lowest glucose concentration observed in a treated pony was 4.1 mM. This was recorded at 8 am, 15.5 h after the last meal was provided, and is within the normal range for horses of 3.4 to 7.4 mM [31]. Furthermore, basal glucose concentrations were not different between control and treated groups. However, glucose concentrations were not assessed after dosing velagliflozin without food provided. In humans, mild or moderate hypoglycemic events have been reported only when SGLT-2 inhibitors have been given in conjunction with concurrent insulin or insulin secretagogues, and the risk of hypoglycemia in humans is considered to be low [32]. Recently, velagliflozin was studied in obese cats and was shown to increase urinary glucose excretion whilst maintaining euglycemia [33]. The authors concluded that in cats the increased urinary glucose excretion was balanced by endogenous glucose production, maintaining normoglycemia.

Another concern about SGLT-2 treatment is its use in subjects with concurrent renal disease. In this study, it would have been useful to measure water intake and urinary output. However, these variables were not quantified due to the technical difficulty of measuring urine output in horses kept under the current conditions. Despite this, no elevation in creatinine or urea was observed in velagliflozin-treated animals after 39 d. It is also worth noting that humans with a defective SGLT-2 gene causing familial renal glucosuria still maintain normal long-term kidney function and the condition does not pose serious physical or clinical consequences for affected individuals [34]. This supports the safety of targeting SGLT-2 as a therapeutic approach. Nevertheless, further studies need to examine the safety and long-term efficacy of velagliflozin in ponies.

Diabetes has been shown to increase the risk of urinary tract infections [35], and with respect to SGLT therapy promoting glucosuria, there are mixed results from human studies regarding an increase in fungal genitourinary infections [36]. No clinical signs of urinary tract infection were observed in the present study, but detailed urinalysis was not performed, although this information would have been useful.

As discussed in our previous paper, a limitation of the diet challenge was that it may not reflect accurately ‘pasture-associated’ laminitis, due to the use of a sweet mixed-feed [12]. However, at 12 g NSC/kgBW/d, the NSC intake from the diet challenge was similar to that obtainable from pasture. With the upper limit of voluntary dry matter intake for a pony being approximately 3% BW [37], a 100 kg pony grazing on pasture that contained 300 g NSC/kg dry matter, could consume up to 9 g NSC/kgBW/d. Thus, our data indicate that while even better results may occur in the field, it would be imprudent not to manage NSC intake carefully in conjunction with velagliflozin treatment, especially in severely dysregulated animals.

It should be noted that the maximum insulin concentration reached during the DCP in 5/12 treated ponies was still above 195 μIU/mL, which appears to have been the threshold beyond which the ponies in this study became at risk of laminitis [12]. The efficacy of velagliflozin in individual ponies might have been influenced by several factors that are clinically relevant. These include the severity of pre-existing insulin dysregulation, the speed of NSC consumption, and the glomerular filtration rate of the kidneys, perhaps influenced by underlying diseases and glucose load.

Further limitations of the present study include the comparatively small sample size of the treated group, the infrequency of monitoring for pathology biomarkers, the lack of measurement of urinary glucose output, and the fact that despite randomisation, the treated group was younger than the control group. The last of these factors could have influenced the outcome if the treated group had better metabolic health before the experiment, but the OGT did not indicate this. Furthermore, within the controls, age had no influence on the development of laminitis. The treated group was smaller than the control group as this was a “proof of concept” study, part of a larger already published study that was designed to investigate laminitis risk in relation to the oral glucose test, and to determine if insulin-associated laminitis could be incited in insulin-dysregulated animals by using a high NSC diet, without causing gastrointestinal side-effects [12].

Additionally, due to difficulty in diagnosing sub or pre-clinical laminitis, it is possible that some ponies that were not diagnosed with clinical laminitis could still have had lamellar pathology, as evidence suggests that subclinical laminitis can occur prior to clinical signs [38]. Euthanasia was not part of the study protocol so lamellar histopathology was unavailable, although it would have helped to confirm the laminitis incidence more precisely.

In summary, both primary aims of the study were accomplished. Velagliflozin improved insulin dysregulation through reducing the insulinemic response to a high NSC diet, and consequently reduced the risk of insulin-associated laminitis. In addition, the compound was well accepted and tolerated, with no adverse effects occurring. Velagliflozin deserves further testing to assess for efficacy and safety in treating insulin-dysregulation over a longer period of time, and also as an acute treatment for insulin-associated laminitis.

## Supporting information

S1 Table Laminitis examination results (median, range) measured on a scale of 0 to 12 before and after a diet challenge period graded in 14 control ponies who developed laminitis; and 23 controls and 12 ponies treated with velagliflozin who did not develop laminitis. (DOCX)

S2 Table Serum insulin and blood glucose concentrations (geometric mean, 95% CI) measured in ponies during a diet challenge period (DCP) over 0, 60, 90, 120 and 240 minutes post-feeding in 11 controls that developed laminitis; 18 controls that did not develop laminitis, and a group of 12 ponies treated with velagliflozin who also did not develop laminitis. (DOCX)

S3 Table Haematology and biochemistry results (mean ± SE) measured before (pre-) and after (post-) a diet-challenge period (DCP) in control ponies that developed laminitis (n = 14); controls that did not develop laminitis (n = 23); and a group treated with Velagliflozin (n = 12) who also did not develop laminitis. Measurements for controls who developed laminitis were taken 4 weeks post-laminitis, whereas measurements for the other groups were taken at the end of the DCP.
(DOCX)

S4 Table Bodyweight (mean ± SE), body condition score and cresty neck scores (geometric mean, 95% CI) measured before and after a diet challenge period (DCP) in control ponies who did not develop laminitis (n = 23) and in ponies treated with velagliflozin (n = 12). (DOCX)
