# Supplementary material for: The sodium-glucose co-transporter 2 inhibitor velagliflozin reduces hyperinsulinemia and prevents laminitis in insulin-dysregulated ponies
Source: PLoS One. 2018 Sep 13;13(9):e0203655. doi: 10.1371/journal.pone.0203655 (PMC6136744; doi:10.1371/journal.pone.0203655)
Supplement: S1 Table — (DOCX) [file pone.0203655.s001.docx]

**S1 Table. Laminitis examination results (median, range) measured on a scale of 0 to 12 before and after a diet challenge period graded in 14 control ponies who developed laminitis; and 23 controls and 12 ponies treated with velagliflozin who did not develop laminitis.**

|  | | **Laminitis**  **(control)** | **No laminitis**  **(control)** | **No laminitis**  **(treated)** |
| --- | --- | --- | --- | --- |
| **Laminitis score** | |  |  |  |
|  | Pre-diet challenge | 0 (0 – 2) | 0.5 (0 – 1.5) | 0.5 (0 – 1.5) |
|  | Post-diet challenge | 5.75 (3.5 – 7.5) | 1 (0 – 3) | 0.5 (0 – 1) |
