# Supplementary material for: The sodium-glucose co-transporter 2 inhibitor velagliflozin reduces hyperinsulinemia and prevents laminitis in insulin-dysregulated ponies
Source: PLoS One. 2018 Sep 13;13(9):e0203655. doi: 10.1371/journal.pone.0203655 (PMC6136744; doi:10.1371/journal.pone.0203655)
Supplement: S2 Table — (DOCX) [file pone.0203655.s002.docx]

**S2 Table. Serum insulin and blood glucose concentrations (geometric mean, 95% CI) measured in ponies during a diet challenge period (DCP) over 0, 60, 90, 120 and 240 minutes post-feeding in 11 controls that developed laminitis; 18 controls that did not develop laminitis, and a group of 12 ponies treated with velagliflozin who also did not develop laminitis.**

|  | | **Laminitis**  **(control)** | **No laminitis**  **(control)** | **No laminitis**  **(treated)** |
| --- | --- | --- | --- | --- |
| **Insulin μIU/mL, time** | |  |  |  |
|  | 0 min | 138 (67 – 284) | 16 (8 – 30) | 14 (6 – 37) |
|  | 60 min | 205 (132 – 320) | 67 (48 – 94) | 51 (30 – 87) |
|  | 90 min | 247 (156 – 392) | 96 (68 – 136) | 68 (39 – 117) |
|  | 120 min | 279 (181 – 430) | 124 (76 – 203) | 93 (52 – 165) |
|  | 240 min | 385 (293 – 505) | 179 (101 – 317) | 140 (88 – 222) |
| **Glucose mM, time** | |  |  |  |
|  | 0 min | 6.2 (4.8 – 7.9) | 4.9 (4.5 – 5.2) | 5.3 (4.7 – 6.1) |
|  | 60 min | 9.7 (7.7 – 12.2) | 7.4 (6.8 – 8.1) | 7.7 (6.8 – 8.6) |
|  | 90 min | 11.8 (9.3 – 14.9) | 8.6 (7.6 – 9.8) | 8.4 (7.3 – 9.7) |
|  | 120 min | 12.5 (9.9 – 15.6) | 9.5 (7.9 – 11.3) | 9.0 (7.4 – 10.8) |
|  | 240 min | 14.8 (12.8 – 17.1) | 9.7 (7.9 – 11.9) | 8.4 (6.7 – 10.8) |
