# Supplementary material for: The sodium-glucose co-transporter 2 inhibitor velagliflozin reduces hyperinsulinemia and prevents laminitis in insulin-dysregulated ponies
Source: PLoS One. 2018 Sep 13;13(9):e0203655. doi: 10.1371/journal.pone.0203655 (PMC6136744; doi:10.1371/journal.pone.0203655)
Supplement: S3 Table — Measurements for controls who developed laminitis were taken 4 weeks post-laminitis, whereas measurements for the other groups were taken at the end of the DCP. (DOCX) [file pone.0203655.s003.docx]

**S3 Table. Hematology and biochemistry results (mean ± SE) measured before (pre-) and after (post-) a diet-challenge period (DCP) in control ponies that developed laminitis (n = 14); controls that did not develop laminitis (n = 23); and a group treated with Velagliflozin (n = 12) who also did not develop laminitis. Measurements for controls who developed laminitis were taken 4 weeks post-laminitis, whereas measurements for the other groups were taken at the end of the DCP.**

|  | **CONTROL (DEVELOPED LAMINITIS)** | | **CONTROL (NO LAMINITIS)** | | **TREATED (NO LAMINITIS)** | |
| --- | --- | --- | --- | --- | --- | --- |
| **Analyte (reference range)** | **Pre-DCP** | **Post-DCP** | **Pre-DCP** | **Post-DCP** | **Pre-DCP** | **Post-DCP** |
| **HCT (0.26 - 0.42)** | 0.37 ± 0.01 | 0.36 ± 0.02 | 0.38 ± 0.01 | 0.38 ± 0.01 | 0.37 ± 0.01 | 0.31 ± 0.02 |
| **RCC (6.0 - 10.5) 10^12^/L** | 7.0 ± 0.3 | 6.8 ± 0.29 | 6.9 ± 0.21 | 7.0 ± 0.22 | 6.9 ± 0.3 | 6.0 ± 0.35 |
| **MCV (36 - 59) fL** | 53.6 ± 0.98 | 53.1 ± 0.95 | 54.8 ± 0.64 | 53.7 ± 0.74 | 54.0 ± 1.04 | 50.8 ± 0.93 |
| **Na (132 - 146) mmol/L** | 136.1 ± 0.69 | 134.4 ± 2.85 | 136.4 ± 0.53 | 137.4 ± 0.40 | 136.0 ± 0.66 | 137.6 ± 0.66 |
| **K (3.1 - 4.9) mmol/L** | 4.0 ± 0.18 | 4.1 ± 0.17 | 4.5 ± 0.22 | 4.2 ± 0.11 | 4.4 ± 0.16 | 4.3 ± 0.15 |
| **Cl (94 - 105) mmol/L** | 98.4 ± 0.55 | 98.7 ± 0.72 | 98.7 ± 0.38 | 99.9 ± 0.36 | 97.7 ± 0.56 | 99.3 ± 0.72 |
| **HCO3 (20 - 32) mmol/L** | 24.1 ± 0.47 | 23.5 ± 1.03 | 23.9 ± 0.52 | 27.9 ± 3.59 | 23.8 ± 0.78 | 24.3 ± 0.38 |
| **An Gap (4 - 19) mmol/L** | 17.7 ± 0.64 | 18.0 ± 0.45 | 18.5 ± 0.35 | 17.1 ± 0.38 | 18.6 ± 0.51 | 18.3 ± 0.43 |
| **Urea (4.4 - 7.8) mmol/L** | 5.2 ± 0.27 | 4.5 ± 0.46 | 5.9 ± 0.28 | 5.2 ± 0.25 | 6.2 ± 0.51 | 5.3 ± 0.32 |
| **Creatinine (55 - 135) umol/L** | 79.1 ± 5.16 | 94.8 ± 5.65 | 80.1 ± 3.15 | 78.9 ± 2.59 | 79.7 ± 3.67 | 73.75 ± 1.84 |
| **Bilirubin (12 - 62) umol/L** | 13.5 ± 1.72 | 13.5 ± 1.72 | 13.4 ± 1.15 | 12.0 ± 0.62 | 14.8 ± 2.29 | 10.8 ± 1.04 |
| **AST (1 - 450) U/L** | 350.1 ± 31.51 | 445.4 ± 35.83 | 345.7± 16.86 | 488.7 ± 43.27 | 393.8 ± 36.47 | 454.9 ± 38.64 |
| **GGT (1 - 49) U/L** | 22.1 ± 2.27 | 38.7 ± 9.70 | 21.9 ± 1.66 | 33.5 ± 5.13 | 25.3 ± 2.96 | 38.8 ± 8.7 |
| **ALP (1 - 280) U/L** | 189.3 ± 16.25 | 143.1 ± 12.97 | 186.7 ± 12.30 | 171.5 ± 7.90 | 230.3 ± 24.15 | 194.2 ± 12.83 |
| **Protein (58 - 72) g/L** | 71.4 ± 1.64 | 77.7 ± 5.5 | 71.6 ± 1.58 | 68. 2 ± 0.87 | 72.2 ± 1.86 | 71.5 ± 2.2 |
| **Albumin (28 - 37) g/L** | 29.4 ± 0.88 | 31.3 ± 1.08 | 29.6 ± 0.73 | 28.6 ± 0.68 | 28.9 ± 0.82 | 30.0 ± 0.82 |
| **Globulins (25 - 42) g/L** | 42.0 ± 1.68 | 41.2 ± 2.75 | 42.0 ± 1.91 | 39.6 ± 1.17 | 43.3 ± 2.16 | 41.5 ± 2.66 |
| **Ca (2.50 - 3.30) mmol/L** | 2.98 ± 0.05 | 3.04 ± 0.07 | 2.98 ± 0.03 | 2.94 ± 0.03 | 2.95 ± 0.11 | 2.98 ± 0.05 |
| **PO4 (0.5 – 1.8) mmol/L** | 1.14 ± 0.08 | 1.27 ± 0.13 | 1.22 ± 0.08 | 1.17 ± 0.07 | 0.99 ± 0.07 | 1.28 ± 0.08 |
| **Creatine Kinase (1 - 648) U/L** | 448.8 ± 54.61 | 494.8 ± 29.38 | 462.7 ± 30.18 | 418.5 ± 26.90 | 455.4 ± 51.02 | 403.4 ± 31.04 |
| **Mg (0.7 - 1.1) mmol/L** | 0.84 ± 0.04 | 0.85 ± 0.05 | 0.79 ± 0.03 | 0.75 ± 0.02 | 0.88 ± 0.07 | 0.77 ± 0.03 |
