# Supplementary material for: The sodium-glucose co-transporter 2 inhibitor velagliflozin reduces hyperinsulinemia and prevents laminitis in insulin-dysregulated ponies
Source: PLoS One. 2018 Sep 13;13(9):e0203655. doi: 10.1371/journal.pone.0203655 (PMC6136744; doi:10.1371/journal.pone.0203655)
Supplement: S4 Table — (DOCX) [file pone.0203655.s004.docx]

**S4 Table. Bodyweight (mean ± SE), body condition score and cresty neck scores (geometric mean, 95% CI) measured before and after a diet challenge period (DCP) in control ponies who did not develop laminitis (n = 23) and in ponies treated with velagliflozin (n = 12).**

|  | | **No laminitis**  **(control)** | **No laminitis**  **(treated)** |
| --- | --- | --- | --- |
| **Bodyweight, kg** | |  |  |
|  | Pre-diet challenge | 215 ± 19 | 219 ± 27 |
|  | Post-diet challenge | 221 ± 20 | 215 ± 25 kg |
| **Body condition score** | |  |  |
|  | Pre-diet challenge | 6.9 (6.4 – 7.4) | 7.4 (6.7 – 8.2) |
|  | Post-diet challenge | 7.1 (6.6 – 7.7) | 7.4 (6.8 – 8.1) |
| **Cresty neck score** | |  |  |
|  | Pre-diet challenge | 3.7 (3.3 – 4) | 3.9 (3.3 – 4.6) |
|  | Post-diet challenge | 4.1 (3.8 – 4.5) | 3.9 (3.3 – 4.5) |
